# New insights into disease‐modifying factors and clinical translation for central nervous system diseases

**DOI:** 10.1002/nep3.70034

**Published:** 2026-03-08

**Authors:** Shen Li, Xunming Ji, Piotr Walczak, Johannes Boltze

**Affiliations:** ^1^ Department of Neurology and Psychiatry, Beijing Shijitan Hospital Capital Medical University Beijing China; ^2^ Beijing Institute of Brain Disorders Capital Medical University Beijing China; ^3^ Chinese Academy of Medical Science & Peiking Union Medical College China; ^4^ Center for Advanced Imaging Research, Department of Diagnostic Radiology and Nuclear Medicine University of Maryland Baltimore Maryland USA; ^5^ School of Life Sciences University of Warwick Coventry UK

**Keywords:** B cells, cholesterol, clinical translation, ischemic stroke, neurodegeneration, neuroinflammation, Parkinson's disease, postconditioning, vagus nerve stimulation, white matter

Disorders affecting the central nervous system (CNS) are often based on the complex interplay of endogenous risk factors including genetic predisposition, comorbidities such as hypertension,^1^ and exogenous risk factors. Moreover, major pathobiological elements and disease‐modifying factors such as peripheral and neuroinflammation significantly contribute to CNS disorders and can modify their course and impact. Augmenting our understanding of disease‐modifying factors is therefore not only crucial for a better comprehension of CNS disorders but will also help to develop novel therapeutic approaches. This includes precision medicine approaches which take the individual disease profile of patients into consideration. Precision medicine approaches are considered as a major step towards more impactful prevention and treatment strategies for cerebrovascular as well as neurodegenerative conditions.^2^, ^3^ Thus, the important role of CNS disease‐modifying factors is increasingly recognized and has been a central subject in previous issues of *Neuroprotection*.^4^ The current issue continues to report discoveries from recent research on neuroinflammation as a CNS disease‐modifying factors. This should help to provide a more comprehensive picture of the current research landscape. The issue also puts a spotlight on clinical translation of experimental treatments with a specific focus on ischemic stroke.

Regulatory B cells (Bregs) are an immunomodulatory and immunosuppressive/anti‐inflammatory B cell subtype counteracting excessive immune responses. A major hallmark of Bregs is the secretion of interleukin (IL)‐10 which can suppress the production of important proinflammatory cytokines such as interferon γ (IFN‐γ), IL‐1, IL‐6, and tumor necrosis factor α (TNF‐α). Thus, Bregs are believed to have the potential of mitigating secondary inflammatory responses which can cause additional brain damage in both acute and chronic neurodegenerative conditions. The review by Stanaszek and Janowski summarizes the current knowledge of Breg actions in the context of CNS disorders. The authors review B cell development and draw a detailed picture of the protective functions of Bregs which are mediated by immunosuppression and anti‐inflammation. They also provide detailed insights into Breg heterogeneity as well as the molecular drivers leading to the development of Breg subtypes. The authors then explain the roles of Bregs in important conditions such as stroke, traumatic brain injury, amyotrophic lateral sclerosis, and multiple sclerosis (MS). They also describe how disturbing the balance of Breg subtypes, especially a potential phenotypic transition into pro‐inflammatory Bregs, may cause detrimental effects in some conditions. The review further provides important suggestions on how future research should explore potential therapeutic applications of Breg cells.

Parkinson's disease (PD) is a chronic neurodegenerative disorder for which no curative treatment exists. Thus, prevention of PD would be a key aspect to decrease overall disease burden, but the causes of sporadic PD are poorly understood. Ajibare et al. present a comprehensive review paper suggesting an interesting concept describing causes that may lead to sporadic PD by summarizing evidence from experimental, mechanistic, and epidemiological studies. Specifically, they suggest a life‐long trajectory with critical periods during which dopaminergic neurons may be more susceptible to damage, providing a foundation for PD. By doing so, Ajibare and coauthors focus on two major pathomechanisms: mitochondrial dysfunction and a proinflammatory environment, again highlighting the role of neuroinflammation in the context of CNS disorders. They also describe processes that may counter neurodegeneration including endogenous neuroplasticity as well as profound physical and cognitive activity. The review not only provides a new perspective and concept, but also suggests numerous testable working hypotheses that, once being addressed experimentally, may augment our pathomechanistic understanding of PD. They also highlight the lack of longitudinal mechanistic data in PD which will be key for future research progress.

High cholesterol levels are a well‐known risk factor of cardiovascular and cerebrovascular conditions as well as diseases to which these contribute. Since many neurodegenerative conditions have a vascular component,^5^ the role of cholesterol metabolism in these conditions is of interest. Kuan et al. present the results of a comprehensive literature review focusing on the role of cholesterol in frequent neurodegenerative conditions including Alzheimer's disease (AD), Huntington's disease (HD), PD, and MS. The review starts by providing an insightful overview of cerebral cholesterol metabolism and its regulation as well as its importance to maintain proper cellular membrane and neurotransmission function. They then explain key aspects of dysregulated cholesterol metabolism in neurodegenerative conditions. The main part of the review focuses on statins, typically being used to treat high cholesterol levels. The analysis reveals that statins do not only provide their protective effects by rebalancing cholesterol levels but also address cholesterol‐independent pathways which have also been reported in the context of ischemic stroke.^6^ Specifically, statins can suppress neuroinflammatory processes and mitigate detrimental microglial responses. Moreover, statins can limit pathobiological processes such as oxidative stress, excitotoxicity, and apoptosis—all of which are important in the context of neurodegenerative disorders. Thus, statins also have the potential to work synergistically with experimental therapies addressing these processes.^7^ Moreover, statins augment the effect of several neurotrophins. The authors then provide detailed insights into evidence from clinical studies reporting beneficial statin effects. The content is meticulously analyzed and presented, so the review provides a detailed overview of high translational importance. Based on this detailed analysis, the authors also provide recommendations on future research direction and critically discuss gaps in as well as limitations of the existing literature.

The review by Jiang et al. focuses on ischemic postconditioning, a promising strategy to mitigate reperfusion injury and other aspects contributing to ischemic tissue damage. Ischemic postconditioning is not only relevant for CNS disorders. It has also been reported to exert protective effects in myocardial, renal, hepatic, and intestinal ischemia. Jiang and coauthors describe the underlying mechanisms with great attention to detail and summarize clinical research activities in these areas. They then focus on ischemic stroke, starting with a comprehensive synthesis of results obtained in small and large animal models. This includes the description of parameters being important for clinical translation of the postconditioning approach, as well as the analysis of safety outcomes. The second part of the review focuses on interventional techniques that can be used clinically to induce ischemic postconditioning in patients with ischemic stroke, emphasizing the importance of endovascular procedures. Current clinical research predominantly focuses on the safety of such procedures and assesses efficacy as a secondary endpoint. Jiang et al. provide a detailed summary of recent clinical trials focusing on both safety and secondary efficacy outcomes. The review concludes by a brief yet profound discussion of translational challenges. The review is supported by comprehensive tables providing a tremendous amount of relevant detail information for interested readers.

Camprubí‐Ferrer and coauthors present results from an experimental study in mice focusing on post‐stroke inflammation and microglial response in the white matter during the recovery phase. Mice were either housed under standard (SE) or enriched environment (EE) conditions. EE is considered an equivalent for intense neurorehabilitation in human stroke patients and has been reported to mitigate stroke impact by improving sensorimotor function.^8^ By using a comprehensive battery of behavioral tests assessing sensorimotor performance, the authors show that EE housing improves and sustains functional recovery, confirming previous reports. Mechanistically, they demonstrate that inflammatory microglial responses in the white matter promotes myelin damage and is positively correlated with infarct size under SE housing conditions. However, these processes are attenuated in mice housed under EE conditions. This suggests that an EE can beneficially modulate post‐stroke inflammation and partially protect against associated myelin damage. Although further studies are needed to fully unravel the underlying mechanisms, comprehensive correlation analyses performed by Camprubí‐Ferrer et al. suggest potential starting points for such downstream studies. The work also establishes white matter microglia as a potential cellular target to foster functional recovery. Given the extended time window of the approach, it would be well compatible with hyperacute cerebroprotection approaches in the context of recanalization therapies^9^, ^10^ both in experimental studies and clinical trials.

Residual functional deficits are a frequent consequence in the chronic phase of ischemic stroke and a major concern in clinical stroke management. Importantly, they are the major reason for the tremendous disease burden, leaving many stroke survivors being dependent on care. Novel approaches to augment the impact of neuroplasticity, ideally also in chronic stroke stages beyond the 6‐month time window of spontaneous and rehabilitation‐assisted functional recuperation are needed to reduce the impact of residual post‐stroke deficits. Yang and coauthors present the protocol of a clinical trial that combines rehabilitation with stimulation of the vagal nerve in chronic ischemic stroke patients enrolled between 9 months and up to 10 years after the ischemic event. The authors review tentative evidence for measurable benefits of the approach which has been obtained in previous clinical trials.^11^, ^12^ They continue by a detailed description of the trial protocol including aspects such as device implantation and stimulation modalities. The outcome measures, including a thorough rationale for their selection, are presented together with the protocol for long‐term analyses of efficacy and safety outcomes. The blinding and randomization procedures which are important elements in this type of clinical trial are also reviewed. The trial is currently in the recruitment phase, so hopefully encouraging results can be reported in the near future.

The current issue of *Neuroprotection* features a mixture of review articles and original contributions focused on disease‐modifying factors as well as recent translational activities in the field. The spectrum of topics is narrower than in previous issues since neuroinflammation is the main disease‐modifying factor discussed. Nevertheless, the featured studies approach different facets of neuroinflammation and also consider other pathobiological elements, thereby providing a holistic and informative picture. Two papers presenting early clinical work in therapeutic approaches are of particular relevance as they provide evidence for the ongoing clinical translation of basic research findings. Thus, we hope that the current issue is of interest to our journal's audience and represents a useful resource when planning own research activities.

In 2026, *Neuroprotection* enters its fourth year of publication and will receive its first impact factor. Indexing in all major databases is completed or about to be completed. Based on secured resources, we can still proudly offer publication services entirely free of charge as a token of gratitude to our community. We foresee that these resources may cover all journal operations at least until the end of the year. In turn, the editorial board is looking forward to receiving insightful contributions from the field of neuroprotection covering most relevant aspects of basic, translational and clinical research. From 2026, *Neuroprotection* will also be open to submissions covering guidelines and recommendations for such research activities.

## AUTHOR CONTRIBUTIONS

**Shen Li:** Conceptualization; data curation; funding acquisition; project administration; resources; software. **Xunming Ji:** Conceptualization; resources; software; supervision. **Piotr Walczak:** Conceptualization; writing—review and editing. **Johannes Boltze:** Con‐ceptualization; data curation; formal analysis; investi‐gation; writing—original draft; writing—review andediting.

## CONFLICT OF INTEREST STATEMENT

X.J., P.W., and J.B. are Co‐Editors in Chief of *Neuroprotection*. S.L. serves as an executive editor for the journal. All authors were blinded from reviewing or making decisions on the manuscript. The article was subject to the journal's standard procedures, with peer review handled independently of the Co‐Editors in Chief and their research groups. P.W. is a founder and holds equity in IntraArt, LLC and Ti‐com, LLC.

## Data Availability

Not applicable.
